# Recovery of mechano-electrical transduction in rat cochlear hair bundles after postnatal destruction of the stereociliar cross-links

**DOI:** 10.1098/rspb.2010.0219

**Published:** 2010-03-31

**Authors:** J. Ebert, S. Fink, A. Koitschev, P. Walther, M. G. Langer, F. Lehmann-Horn

**Affiliations:** 1 Institute of Applied Physiology, Ulm University, Albert-Einstein-Allee 11, 89081 Ulm, Germany; 2 Institute of Electron Microscopy, Ulm University, Albert-Einstein-Allee 11, 89081 Ulm, Germany; 3 THRC Tuebingen Hearing Research Center and Department of Otorhinolaryngology, Tuebingen University, Tuebingen, Germany

**Keywords:** rat, organ of Corti, outer hair cells, tip link recovery, mechano-electrical, transduction

## Abstract

Mechano-electrical transduction (MET) in the stereocilia of outer hair cells (OHCs) was studied in newborn Wistar rats using scanning electron microscopy to investigate the stereociliar cross-links, Nomarski laser differential interferometry to investigate stereociliar stiffness and by testing the functionality of the MET channels by recording the entry of fluorescent dye, FM1-43, into stereocilia. Preparations were taken from rats on their day of birth (P0) or 1–4 days later (P1–P4). Hair bundles developed from the base to the apex and from the inner to outer OHC rows. MET channel responses were detected in apical coil OHCs on P1. To study the possible recovery of MET after disrupting the cross-links, the same investigations were performed after the application of Ca^2+^ chelator 1,2-bis(o-aminophenoxy)ethane-*N*,*N*,*N*′,*N*′-tetraacetic acid (BAPTA) and allowing the treated samples to recover in culture medium for 0–20 h. We found that the structure and function were abolished by BAPTA. In P0–P1 samples, structural recovery was complete and the open probability of MET channels reached control values. In P3–P4 samples, complete recovery only occurred in OHCs of the outermost row. Although our results demonstrate an enormous recovery potential of OHCs in the postnatal period, the structural component restricts the potential for therapy in patients.

## 1. Introduction

Cochlear hair cells are highly specialized for transducing the slightest movements of the tectorial membrane into electrical signals that are transported along the acoustic nerve to the brain. The cells are arranged in three rows of outer hair cells (OHCs) and one row of inner hair cells (IHCs). The apical portion of each cell consists of an arrangement of densely linked protrusions, which form the typical stereocilia. The stereocilia are also linked by fine filaments, and those linking the tips of the stereocilia with one another (the tip links) are assumed to be a crucial prerequisite for the stereociliar mechano-sensitive complex to function. Stereociliar tips contain transmembrane channels that conduct an ion current when movement of the tectorial membrane increases the mechanical tension in the tip link and stereociliar membrane, which is referred to as mechano-electrical transduction (MET). Movement in the opposite direction decreases stereociliar membrane and tip-link tension, thereby closing the MET channels that are open at rest.

Little is known about the maturation of MET channel function in newborns. Evidence from mouse vestibular hair cells indicates that MET begins with an all-or-none onset between embryonic days 16 and 17. The functional MET channel complex is possibly first assembled at the base of the stereocilia and then migrates, driven by myosin 1c molecules, with a cross-link in tow to the stereociliar tips. When the cross-link has reached the tip of the shorter stereocilium, its other side continues to climb until sufficient tension is developed (Geleoc & Holt 2003).

Although rats and mice do not begin hearing before postnatal days 11–12, as measured by evoked auditory potentials, the stereocilia already express ion channels during embryonic development. Ion currents, though not conducted by the MET channels, can be registered in the faster developing basal coil IHCs of the mouse on embryonic day 14.5; in the somewhat slower apical coil IHCs, the currents appear on embryonic day 15.5 (Marcotti *et al*. 2003; Helyer *et al*. 2005).

The onset of MET currents in the basal coil IHCs of the mouse has been shown electrophysiologically to occur on the day before birth. FM1-43, a styryl dye that rapidly and specifically labels hair cells and permanently blocks MET channels, was shown to enter the cells at the same time. In mouse OHCs, MET currents have been detected at the base from the day of birth (P0) onwards. During the days following birth, the ability of the stereocilia to conduct MET currents extends towards the apex (Lelli *et al*. 2009). Similarly, apical OHCs from rats are not able to conduct MET currents when the animals are dissected before P2 (Waguespack *et al*. 2007).

The general aim of the present study was to comprehensively evaluate the MET mechanism in OHCs from the rat cochlea. Of special interest was a comparison between cross-linking filament density and hair-bundle structure and stiffness and the relationship of these parameters with the functionality of the MET channels.

In particular, though, we wanted to study the ability of the transduction mechanism to recover from a temporary loss of all stereociliar cross-links as this information, though known for several non-mammalian species, is not yet available for mammals. 1,2-Bis(o-aminophenoxy)ethane-*N*,*N*,*N*′,*N*′-tetraacetic acid (BAPTA), a potent calcium chelator, was used to disrupt the cross-links, particularly the tip links, as this has been shown to result in MET channel closure in both mammals and non-mammals (Assad *et al*. 1991; Goodyear & Richardson 1999, 2003; Kachar *et al*. 2000; Rzadzinska *et al*. 2004; Goodyear *et al*. 2005; Sellick *et al*. 2007). However, closure of the MET channels following a tip-link loss had been questioned by one group (Meyer *et al*. 1998, 2005). The effect of BAPTA was elucidated by the finding that cross-links form a complex with calcium-dependent cadherin 23 (CDH23) and protocadherin 15 (Kazmierczak *et al*. 2007). During ontogenesis, CDH23 is a component of the cross-links (Siemens *et al*. 2004) as well as transient lateral links (Michel *et al*. 2005), both of which are sensitive to low calcium concentrations (Kazmierczak *et al*. 2007). The tension sustained by the tip links and the adaptation of the MET channels are both assumed to be regulated by myosin 1c molecules (Holt *et al*. 2002; Gillespie & Cyr 2004; Stauffer *et al*. 2005).

Because stereocilia of the rat cochlea undergo rapid structural and functional changes after birth, we used samples dissected immediately after birth (P0). We also used samples from older (P3–P4) animals for comparisons.

## 2. Material and methods

We investigated the morphological, mechanical and functional changes in rat OHC bundles after postnatal disruption of the interstereociliar cross-links by BAPTA. The changes in hair-cell bundle morphology, especially the cross-links, were studied using scanning electron microscopy (SEM). Alterations in hair-bundle stiffness were measured using Nomarski laser differential interferometry (DIF). The functionality of MET channels was tested by measuring the entry of fluorescent dye, FM1-43, in hair-cell bundles. All experiments were performed at room temperature (22–25°C).

### (a) Dissection of the organ of Corti

The organs of Corti from newborn to 4-day-old (P0–P4) Wistar rats (Charles River GmbH, Sulzfeld, Germany) were prepared as reported previously (Sobkowicz *et al*. 1975; Russell & Richardson 1987). In brief, the coiled organ of Corti was isolated, put in HEPES-buffered solution (concentrations in mM: 5.4 KCl, 0.5 MgCl_2_, 0.4 MgSO_4_, 141.7 NaCl, 1.6 CaCl_2_, 10.0 HEPES, 3.4 l-glutamine and 6.3 d-glucose dissolved in pure water, pH 7.4), and divided into three C-shaped parts: the basal, medial and apical turn. As a matter of course, the apical turns, and on a few occasions the medial turns, were used for experimentation. The pieces were immobilized on glass coverslips with either small borosilicate glass fibres (diameter 150–250 µm) for electron microscopy and hair bundles stained with FM1-43 (Invitrogen Molecular Probe, Karlsruhe, Germany), or celltak (BD Biosciences, Two Oak Park, Bedford, MA) for DIF. Prior to investigation, the samples were kept for 1–4 h in dishes filled with 3 ml culture medium (MEM D-VAL; Gibco BRL, Paisley, UK) supplemented with 10 per cent heat-inactivated FCS and 10 mM HEPES buffer (pH 7.2) at 37°C and 5 per cent CO_2_. No antibiotics were added to the medium. For experiments, a sample was transferred to standard saline solution containing (in mM) 144.0 NaCl, 0.7 NaH_2_PO_4_, 5.8 KCl, 1.3 CaCl_2_, 0.9 MgCl_2_, 5.6 d-glucose and 10.0 HEPES–NaOH (pH 7.4).

### (b) Disruption of stereociliar links by BAPTA

For disruption of the interstereociliar filaments, the samples were bathed for 30 min in standard saline depleted of calcium by the addition of 5 mM BAPTA. The samples were then returned to culture medium and examined immediately (0 h), 6 or 20 h after BAPTA treatment. In the text, ‘days’ refer to the age of the animal on the day of dissection, and ‘hours’ refer to the time passed between BAPTA treatment and experimental investigation (SEM, DIF or fluorescence microscopy of FM1-43 staining).

### (c) Structural measurements using SEM

Following incubation, the BAPTA-treated and sham-treated (control) samples were quickly rinsed in 0.25 M HEPES buffer solution and chemically fixed for at least 4 h at room temperature using 2.5 per cent glutaraldehyde in 0.1 M HEPES buffer containing 2 mM CaCl_2_ (pH 7.2) The samples were then dehydrated in a graded ethanol series, dried in liquid CO_2_ and mounted on specimen holders with double-sided carbon sticky tape. The samples were rotary sputter-coated with platinum–carbon (2 nm thick). A Hitachi S-5200 in-lens field emission SEM (Hitachi Limited, Tokyo, Japan) was used at 3–10 kV. Twenty-nine BAPTA-exposed samples from P0 rats, 19 BAPTA-exposed samples from P3–P4 rats and 43 samples from non-BAPTA-exposed control rats (*n* = 22 P0 and *n* = 21 P3–P4) were investigated. The number of links was counted in at least two samples for each condition. Top views of single hair bundles were studied in samples from P0 rats, whereas side views were used to make lateral links better visible in samples from P3–P4 rats.

### (d) Hair-cell bundle stiffness measured by laser DIF

Stiffness measurements were performed optically by investigating the Brownian motion of the hair-cell bundles. A focused, low-power laser beam (15 mW at 632.8 nm; Schäfter und Kirchhoff, Hamburg, Germany) with mono-mode fibre optics (F810FC-635 FC/PC Collimation Pkg/420–650 nm, AR-coated; Thorlabs, Dachau, Germany) added to a differential interference contrast microscope (Axioskop 2 FS Plus; Zeiss, Oberkochen, Germany) with a sensitivity of 1 pm/square root of the frequency was used. The beam was focused onto the stereocilia with a water-immersion objective (Achroplan ×40, numerical aperture = 0.80; Zeiss). The collimator of the microscope collected the divergent rays. Two orthogonally polarized laser beams separated by approximately 0.2 µm at their foci illuminated the stereocilia. Slight movement of the object from one beam towards the other caused a phase shift between the transmitted rays and intensity modulation at a quadrant detector (SD 085-23-21-021; Advanced Photonix, CA) where both rays interfered.

The signals were digitized by an AD-converter board (Instrutech, ITC-18, Lambrecht/Pfalz, Germany) and recorded with the custom-made ScanClamp 0.89 software (Universities of Tuebingen and Ulm, Germany). Fourier transformation, averaging and power-spectrum calculations, from which stiffness was derived, were performed using Igor Pro 4.09A Carbon (Wave Metrics, Lake Oswego, OR). We assumed that false-high stiffness values were measured at some hair bundles that deviated from the direction of the laser beam. To reduce the effect of these measurements, we calculated the medians and first and third quartiles (Microsoft Excel X Mac Service Release 1). Significance was determined by the student's *t*-test and set at *p* = 0.05. An intact morphology is essential; thus, some hair-cell bundles were examined by SEM immediately following the measurements. Additional details concerning DIF were published previously (Denk *et al*. 1989).

### (e) Detection of open MET channels using FM1-43

BAPTA-treated hair cells from P0 (*n* = 15) and P3–P4 (*n* = 11) rats were loaded in 3 µM FM1-43 for 10 s and kept for 20 min in standard solution prior to examination. Light from a mercury lamp (HBO 100W/2; Zeiss) passed through a bandpass filter (filter set 13/bandpass 470/20) illuminated the samples. Emitted light above 590 nm was detected (filter set 15/longpass 590 nm). A beam splitter (filter set 15/FT 580, all from Zeiss) was used, separating the excited wavelength of light from the emitted light. For all conditions, the fluorescence images were generated with the same CCD-camera (Spot Insight Monochrome; Diagnostic Instruments, Sterling Heights, MI, USA) settings. Final images were processed using Adobe Photoshop 7.0 (Adobe Systems Incorporated, San Jose, CA, USA). Sham-treated (without BAPTA) P0 (*n* = 10) and P3–P4 (*n* = 9) samples served as controls.

In order to determine whether the dye entered the hair cells through open MET channels, additional control experiments were performed with samples kept for 20 min in standard solution containing 100 µM of the channel blocker dihydrostreptomycin (DHSM) before application of the dye and successive fluorescence imaging.

## 3. Results

### (a) Morphology of apical OHC bundles

#### (i) Normal development

**Figure 1.**
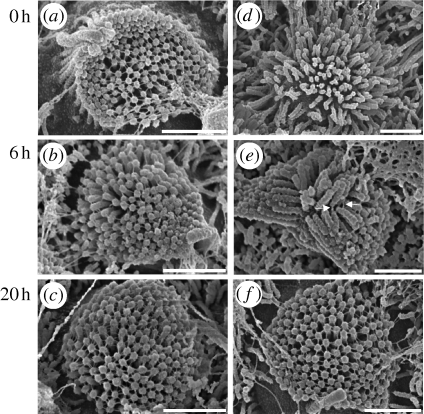
Scanning electron microscopic top views of apical OHC bundles dissected from the organs of Corti of rats sacrificed on their day of birth (P0). (*a–c*) Development: controls after 0, 6 or 20 h in culture medium. (*d–f*) Recovery after BAPTA. BAPTA-treated bundles. (*d*) Immediately after treatment, all links disappeared and the bundles were disorganized with splayed stereocilia. (*e*) After 6 h, links connecting neighbouring adjacent stereocilia at their bases (arrows) reappeared. (*f*) After 20 h, various types of links connected adjacent stereocilia, similar to controls. Scale bar, 1.0 µm.

**Figure 2.**
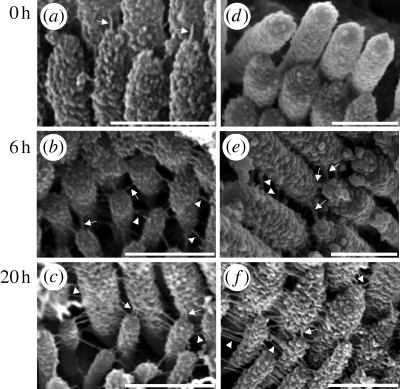
Scanning electron microscopic top views of apical OHC bundles dissected from the organs of Corti of rats sacrificed 3–4 days after birth (P3–P4). (*a–c*) Development: controls (*d–f*) Recovery after BAPTA: BAPTA-treated bundles. (*d*) Immediately after treatment, both lateral links and tip links were disrupted but no stereociliar splaying was seen. (*e*) After 6 h and (*f*) 20 h of recovery in culture medium, numerous tip links (arrows) and lateral links (arrowheads) reappeared. Scale bar, 0.5 µm.

High-resolution SEM images of apical hair-cell bundles taken on P0 revealed stereocilia arranged in a round brush-like manner (figure 1*a*–*c*). The stereocilia all appeared to be roughly the same height. Stereocilia were connected by multiple links of various orientations. No morphological changes were observed when culture was extended from 6 to 20 h (figure 1*b*,*c*). The bundles in P3–P4 samples were V-shaped with at least three to four rows of stereocilia in graduated heights (figure 2*a*–*c*). Two types of link were distinguishable: (i) tip links connecting the tip and shaft of adjacent stereocilia from different rows (figure 2*b*, arrows) and (ii) lateral links connecting stereocilia horizontally at their shafts (figure 2*b*, arrowheads).

#### (ii) Recovery of links after disruption by BAPTA

BAPTA treatment removed all links in P0 samples, which resulted in disorganized hair bundles with splayed stereocilia (figure 1*d*). Six hours later, new links had grown, connecting adjacent stereocilia at their bases (figure 1*e*, arrows). The number of links was 0.5 per stereocilium (*n* = 7; number of observed hair bundles with SEM). After 20 h, the links had moved to the tips of the stereocilia. The morphology of the hair-cell bundles appeared normal with erect stereocilia of nearly uniform height. The number of connecting links had increased to 1.43 per stereocilium (*n* = 10), which was in the range of controls (1.01 for controls acutely dissected and fixed, *n* = 10; 1.22 for controls kept in culture medium for 6 h, *n* = 7; 1.52 for controls kept in culture for 20 h, *n* = 9).

In samples from P3–P4 animals (figure 2*d*–*f*), BAPTA destroyed all types of links almost completely without major splaying of the stereocilia (figure 2*d*). Six hours later, the tip links and lateral links had recovered. Interestingly, multiple tip links were found in control hair bundles (figure 2*b*,*c*) and after recovery from BAPTA treatment (figure 2*e*,*f*). After 6 h, the number of recovered tip and lateral links per stereocilium was 0.37 and 0.93 (*n* = 9), respectively. In controls (*n* = 4), after 6 h of development, 0.53 tip links and 1.54 lateral links were counted per stereocilium.

When recovery was examined after 20 h, the number of tip links increased to 0.51 and the number of lateral links to 1.17 (*n* = 7). These values were still smaller than those of controls (0.63 for tip links and 1.53 for lateral links, *n* = 5).

### (b) Stiffness of apical and medial OHC bundles

#### (i) Normal development

**Figure 3.**
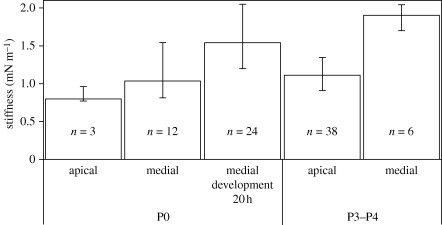
Stiffness of OHC bundles during development. The median was determined for apical and medial cochlear preparations. Note that the stiffness increased with postnatal age, and even in P0 preparations after 20 h in culture. The error bars represent the first and third quartiles, *n,* number of investigated hair bundles.

Hair-cell bundle stiffness significantly increased from 0.80 to 1.12 mN m^−1^ (apical, *p* = 0.035) and 1.04 to 1.90 mN m^−1^ (medial, *p* = 0.013) within the first postnatal days (P0 versus P3–P4; figure 3). Follow-up investigations of the stiffness of P0 preparations (medial turn) showed a significant increase in stiffness after 20 h of culture to almost 150 per cent the value of non-cultured (P0) hair bundles (*p* = 0.013). No significant difference was measured between P0 medial hair bundles cultured for 20 h and non-cultured P1 medial hair bundles with a median stiffness of 1.35 mN m^−1^ (data not shown).

The stiffness of apical hair-cell bundles from P3–P4 rats was significantly less than the stiffness of the medial bundles from the same group of rats (*p* = 0.023). This finding is in agreement with other reports that the cochlear base precedes the apex in development (Ruben 1967; Lanford *et al*. 1999; Waguespack *et al*. 2007).

#### (ii) BAPTA-induced disruption and recovery of connecting links

**Figure 4.**
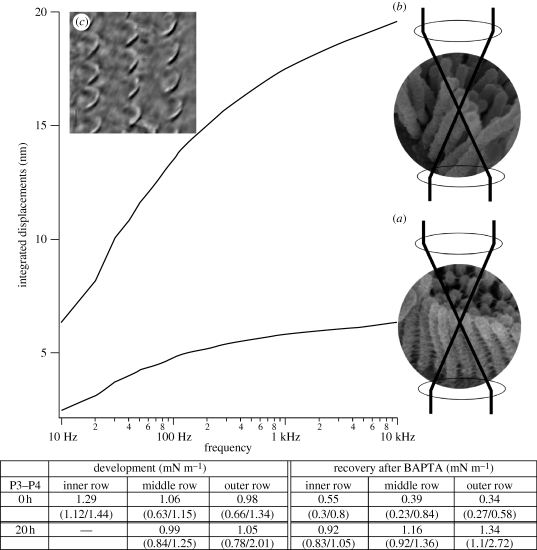
Representative integrated spontaneous (Brownian) motion of the same P3–P4 outer apical hair cell bundles before (lower curve) and after (upper curve) the BAPTA-induced loss of links. For each trace, the Fourier transformation was calculated from 10 000 ms of raw data on recorded displacement. The power spectral densities measured at the cuticular plate were subtracted from the power spectral densities measured at the stereociliar tips before integration, making the background noise and movements of the hair-cell bodies irrelevant. Insets: (*a*) control hair bundle from P4 with interfering laser beams and (*b*) after BATPA interfering laser beams. (*c*) Laser intensity demonstrated as a greyscale image composed of 50 line scans of the hair bundles in the three rows of OHCs. The table displays the median stiffness values for the apical OHCs on P3–P4 after BAPTA-induced loss of links and 20 h of recovery. The first and third quartiles are in brackets.

The Brownian motion of control hair bundles (P3–P4) in a fluid constituted a damped oscillation, the amplitude increasing with frequency up to approximately 1 kHz, at which the maximal displacement was less than 7 nm (figure 4, lower trace). After BAPTA-induced disruption of the connecting links, maximal displacement increased up to almost threefold in the same hair bundle (figure 4, upper trace). Because measuring stiffness before and after regeneration is not technically possible in the same hair bundle, we were forced to calculate average values. Corresponding to increased displacement, the stiffness of the hair-cell bundles decreased significantly after BAPTA treatment. The stiffness of the inner row of OHC bundles decreased to 43 per cent (*p* = 0.008) of control values, and the stiffness of the middle and outer rows of apical OHC decreased to 37 (*p* = 0.017) and 35 per cent (*p* = 0.001), respectively. After 20 h of recovery, the stiffness significantly increased in the outer (*p* = 0.002) and middle (*p* = 0.003) rows to values that did not significantly differ from control, and the inner row only showed a tendency to regain its former stiffness (*p* = 0.084).

### (c) Functionality of MET channels

Intact tip links are required for normal MET channel operation. At rest (no forces applied to the hair bundle), MET channels have an open probability of roughly 20 per cent in frog saccular hair cells (Jaramillo & Wiesenfeld 1998). An ‘excitatory’ bundle deflection increases the tension in the tip links, thereby increasing the open probability of the channel. Using FM1-43 as an indicator of the functionality of MET channels in apical coil OHCs, we found that open channels were already present on the first day after birth.

**Figure 5.**
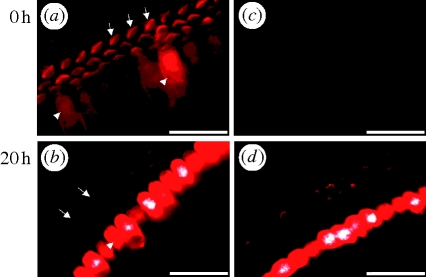
Recovery of the ability of FM1-43 to stain P1 apical coil hair cells. (*a*,*b*) Development: control with V-shaped hair bundles of the OHCs (arrows) and cell bodies of IHCs (arrowheads) labelled. (*c*,*d*) Recovery after BAPTA. (*c*) Link disruption by BAPTA abolished the influx of FM1-43 into IHCs and OHCs. (*d*) Hair bundles recovered MET channel function after a period of recovery, leading to FM1-43 influx. Scale bars, 20 µm.

In apical P1 samples exposed to BAPTA, no FM1-43 signal was detected, indicating that the MET channels were closed or non-functional (figure 5*c*). After 20 h of recovery in culture, fluorescence was detected (figure 5*d*). Together with the results obtained for morphology and stiffness, this observation indicates that restored tip links had functional MET channels. All control P1 hair bundles exposed to the dye had a clearly detectable fluorescence signal in both IHCs and OHCs (figure 5*a*,*b*). FM1-43 stained none of the supporting cells, indicating that the dye does not enter cells without MET channels. The same results were obtained in medial OHCs and IHCs at P1 and in apical and medial coil hair bundles at P3 (data not shown).

To test whether the dye entered the cells via cell membrane leaks, which might have arisen during the dissection or recovery period, we performed experiments blocking the MET channels with DHSM (Marcotti *et al*. 2005) prior to FM1-43 application. Under these conditions, the dye should not be able to enter the hair cells, and we did not detect any fluorescence (data not shown).

## 4. Discussion

The results show that all cross-links in apical OHCs of the mammalian cochlea are both structurally and functionally destroyed by the application of BAPTA. Using samples taken from P0 rats, we found that the cross-link destruction was reversible and MET was capable of recovering completely. Restoration of tip-link numbers did not reach full control values in cells from P3 and P4 rats. However, functional recovery occurred to some extent. Complete recovery was restricted to the outermost rows of OHCs, even when the samples were allowed to recover for 20 h. Thus, the self-repairing transduction apparatus seems to persist during the early process of functional maturation, which is positive with respect to clinical hopes. This might explain why exposure of adults to loud sound causes threshold shifts that are only temporary. However, a massive trauma that not only breaks tip links but also damages the overall structure of the hair bundles results in permanent loss of function although the tip links may recover.

Nevertheless, the finding that mammalian OHCs at a very early developmental stage recovered from the effects of BAPTA treatment is promising. In addition, the findings support the existing hypothesis that cross-links are essential for the formation and preservation of the hair-bundle structure during maturation.

### (a) Structural findings

Our SEM results show that the stereociliar arrangement of cochlear hair cells in very early developmental stages is very sensitive to cross-link disruption. The disorder of the stereociliar arrangement upon BAPTA treatment suggests that, in the early stages, the stiffness of the stereociliar actin cytoskeleton is very low. In contrast, in more mature hair cells, the structural arrangement of stereocilia remained intact upon BAPTA treatment, suggesting that the actin cytoskeleton substantially contributes to overall stereociliar stiffness. Obviously, the stability of the structural arrangement of the stereocilia is ensured at all stages of development.

### (b) Stiffness values during normal development

Our results with control preparations can be compared with results obtained in previous research in which the stiffness of mammalian sensory hair bundles was measured by other methods. Using a calibrated glass fibre, Russell *et al*. (1992) determined the stiffness in apical outer hair bundles of P1–P2 mice to be 1.42 mN m^−1^, whereas Langer *et al*. (2001), using AFM sensors on OHCs from P4 rats, found the stiffness to be 2.5 mN m^−1^ in the excitatory and 3.1 mN m^−1^ in the inhibitory direction. Using water jet deflection of OHC bundles from P1–P2 mice, Geleoc *et al*. (1997) calculated the stiffness to be 4.5 mN m^−1^.

Compared with these data, our results of 0.8 mN m^−1^ in P0 apical OHCs and 1.9 mN m^−1^ in P3–P4 medial OHCs are in the lower range for either stage of development. This difference was probably caused by our use of a non-contact, passive laser DIF method, measuring the Brownian motion around the resting position and not inducing any kind of adaptation.

The DIF method has already been applied to saccular hair-cell bundles from the bullfrog. These measurements also resulted in stiffness values (0.35 mN m^−1^) (Denk *et al*. 1989) that were lower than those obtained with glass fibres (ranging from 0.8 to 1.1 mN m^−1^) (Marquis & Hudspeth 1997; Martin *et al*. 2000). For long stereocilia, the bullfrog's saccular hair bundles were found to have even lower stiffness values than our mammalian OHCs.

### (c) Change in stiffness during maturation

Little is known about the changes in hair-bundle stiffness during maturation. Two counteracting processes are supposed to occur; the number of interstereociliary links declines progressively, but the number and density of intra-stereociliar actin filaments increase (Tilney & DeRosier 1986).

The organ of Corti and MET properties develop similarly *in vitro* and in appropriate acute preparations (Waguespack *et al*. 2007). In our experiments with medial OHCs from P0 rats, the stiffness increased to nearly 150 per cent of the original value when the samples were kept in culture for 20 h. This finding indicated normal development because the stiffness of medial OHCs from P3–P4 rats was 182 per cent the stiffness found in P0 rats.

The increase in overall hair-bundle stiffness with maturation suggests that the interconnecting links contribute to a larger portion of the overall bundle stiffness in early stages of development and make up the structural integrity of the hair bundle. The actin cytoskeleton of the stereocilia and the side links gain functional importance over time by generating hair-bundle stiffness, whereas the tip links become increasingly important for the transduction process. This hypothesis is supported by our SEM images showing strong BAPTA-induced damages in immature hair bundles (P0), but hardly recognizable morphological changes in further developed (P3–P4) bundles.

Our finding that the stereocilia of the innermost row of OHCs have greater stiffness than those in the middle and outer rows suggests that stereociliar development is not only graded from base to apex (Ruben 1967; Lanford *et al*. 1999; Waguespack *et al*. 2007), but also from inside to outside. Despite this functional difference detected by DIF at the same point along the cochlea, SEM observations did not reveal a difference in maturation between the three rows of OHCs. Thus, it appears that DIF is a very sensitive method for detecting local differences in stiffness.

### (d) Recovery of stiffness following the disruption of links by BAPTA

Marquis & Hudspeth (1997) showed that BAPTA treatment of bullfrog saccular hair bundles reduces their stiffness by approximately 80 per cent. This finding led them to conclude that roughly 80 per cent of the stiffness of a hair bundle is due to connecting links. We found that exposing apical OHCs from P3–P4 rats to BAPTA reduced their stiffness by only 57–65%. This discrepancy could mean that, in mammals, the links have a smaller effect on the overall hair-bundle stiffness than in lower vertebrates.

The Brownian motion of a single stereocilium was three times higher than controls after the connecting links were destroyed by BAPTA, but the newly developed links seemed to be as mechanically effective as the original links after recovery because BAPTA-treated hair bundles allowed to recover in culture medium had roughly the same stiffness as sham-treated controls. This led us to speculate that the reappearance of tip links in SEM images might signify that the MET channel complex might also be operational.

Interestingly, the hair bundles of the outermost row of OHCs were stiffer after recovery than the bundles of the middle and inner rows, which could be an additional sign of the strong developmental dependence of the rebuilding process. Such a dependence of the functional recovery on the developmental stage has already been shown in P1–P2 mice, with FM1-43 loaded cells showing a gradient along the organ of Corti (Gale *et al*. 2001).

Links seem to be important for the development of the bundle's typical shape at an early postnatal stage. After disruption of the links, the stereocilia become disorganized on P0, as demonstrated by SEM. Recovered links somehow manage to erect the splayed stereocilia again. Recovered links may contribute to active mechanisms for approaching stereociliar tips. Such an approach could be managed by the myosin molecules that are normally found at the end of the tip links and are usually responsible for adaptation processes. A mechanism for recovery could be the migration of basal MET complexes to stereociliar tips, driven by myosin molecules. Such a mechanism is described for the onset of transduction in embryonic mouse vestibular hair cells (Geleoc & Holt 2003).

Supporting this hypothesis, SEM images taken after 6 h of recovery showed unspecified links connecting stereociliar shafts at their bases. Twenty hours after BAPTA treatment, the OHCs of P0 and P3–P4 rats were morphologically similar to controls, with unspecified links in P0 animals and interconnecting links, including tip links and lateral links, in P3–P4 animals. The recovery of links in P0 animals seems to proceed at a slower pace than in P3–P4 animals, with connecting links already located at the stereociliar tips after 6 h of recovery.

However, the number of recovered links was lower for both P0 and P3–P4 hair bundles, compared with controls, 6 h after BAPTA-induced link loss. After 20 h of recovery, the number of links almost reached control levels in P0 samples. In contrast, the number of tip and lateral links in P3–P4 samples was lower than in controls. Because the number of visible lateral links depends on the SEM perspective, these results are not significant, but are in agreement with a reduced recovery potential for more developed hair bundles, as shown in stiffness measurements. Furthermore, it is more difficult to investigate the recovery of all kinds of links with maturation owing to growing stiffness by increasing the number of intracellular actin filaments. The cytoskeleton of the hair cells is less flexible and preparations become more sensible to intra- and intercellular disruption, such as disruption of interstereociliar links.

### (e) MET channel functionality

Short exposure to the fluorescent dye FM1-43 selectively labels the sensory hair cells in the organ of Corti, as has been shown for zebrafish (Seiler & Nicolson 1999), bullfrog sacculi (Gale *et al*. 2000) and the cochlear hair cells of the mouse (Gale *et al*. 2001; Meyers *et al*. 2003). In preparations from mammals, FM1-43 was shown to enter hair cells through open MET channels and block them permanently (Gale *et al*. 2001; Meyers *et al*. 2003). MET channels adopt the closed state after disruption of the tip links by BAPTA, eliminating FM1-43 labelling as a valid test for normal operation of the transduction mechanism. In our experiments, FM1-43 no longer stained the OHCs after they were treated with BAPTA, but the staining returned when the cells were allowed to recover. We interpret this observation as a sign of MET channels regaining functionality.

MET channel responses in mouse basal coil OHCs can already be detected in P0 preparations, whereas the responses in apical coil OHCs are only seen when the animals reach P2 or later (Lelli *et al*. 2009). Also, for rat apical coil OHCs, MET channel responses are not detected earlier than P2 (Ruben 1967; Lanford *et al*. 1999; Waguespack *et al*. 2007). In contrast, our results with apical P1 cochlear hair bundles suggest the presence of functional MET channels on P1. The earlier response of rats can be explained by the different methods; we measured the spontaneous uptake of FM1-43 without hair-bundle deflection, whereas other studies measured the MET current following mechanical deflection of the hair bundles. Because the links have no unidirectional structure on P1, unidirectional mechanical deflection might not be capable of creating an MET current. We found the fluorescence of apical OHCs and IHCs on P1 to be more intense after 20 h of incubation compared with immediately after dissection, suggesting a rapid increase in operational MET channels within hours (figure 5*a*,*b*). Because this process was superimposed by quenching the fluorescent dye, we did not provide quantitative figures.

## 5. Conclusion

Our results demonstrate that cross-links are major contributors to the overall stiffness of cochlear hair bundles at all stages of development (at least P0–P4). In particular, the circular arrangement of the stereocilia and a dense network of radially oriented cross-links seem to promote the structural recovery of damaged hair bundles. However, high actin polymerization activity as observed during the development of the inner ear seems critical for the recovery of hair-bundle structure after damage.

These latter conditions might explain why the mammalian cochlea shows a permanent threshold shift after exposure to noise at high sound pressure levels. Stereociliar hair bundles in adult cochlear hair cells also do not show a circular arrangement or a high number of cross-links. Additionally, high polymerization activity is very unlikely in the adult hair bundles of the adult mammalian cochlea. However, we show that the MET channels are able to recover at all stages of development, up to P4.
